# Effects of Long-Term Supplementation with Brown Seaweeds and Polyphenols in Rabbit on Meat Quality Parameters

**DOI:** 10.3390/ani10122443

**Published:** 2020-12-20

**Authors:** Raffaella Rossi, Francesco Vizzarri, Sabrina Ratti, Marisa Palazzo, Donato Casamassima, Carlo Corino

**Affiliations:** 1 Dipartimento di Medicina Veterinaria, Università Degli Studi di Milano, Via dell’Università 6, 26900 Lodi, Italy; sabrinaratti@hotmail.com (S.R.); carlo.corino@unimi.it (C.C.); 2 Department of Agricultural and Environmental Science, Università di Bari Aldo Moro, Via G. Amendola 165/A, 70126 Bari, Italy; francesco.vizzarri@uniba.it; 3 Department of Agricultural, Environmental and Food Sciences, Università Degli Studi del Molise, Via F. De Sanctis 1, 86100 Campobasso, Italy; m.palazzo@unimol.it (M.P.); casamassima.d@unimol.it (D.C.)

**Keywords:** brown seaweed, meat quality parameters, plant polyphenols, rabbit

## Abstract

**Simple Summary:**

Safe and natural feed supplement are required to enhance animal health and welfare, answering to the agri-food system and consumers’ needs. The research for sustainable feed additives for enhancing rabbit health and meat quality parameters are needed. Plants received considerable consideration as safe feed supplements in animal nutrition. Recent studies indicated that seaweed can be considered a sustainable dietary supplement that improve animals’ health and meat quality parameters. Moreover, plant polyphenols have been studied as antioxidants and cholesterol-lowering agents in meat. No data on the effects of long term dietary supplementation of a mixture composed of brown seaweed and plant polyphenols has been available. For this reason, we decided to test the effects of dietary natural supplements in rabbits, based on performance and meat quality parameters.

**Abstract:**

The objective of the present study was to evaluate the effects of dam and offspring dietary supplementation with a natural feed additive on the growth performance and meat quality parameters of growing rabbits. The growing rabbits are selected from lactating does receiving a control diet (C) or diets supplemented with 0.3% (SP1) and 0.6% (SP2) of feed additive containing brown seaweeds (*Laminaria* spp.) and plant extracts. In the postweaning phase, the growing rabbits remained in the treatment group defined by their does and the trial lasted 42 days. The average daily feed intake and feed conversion ratio were improved in the rabbit fed 0.6% of the natural feed additive. The cholesterol content tended to be lower in *Longissimus lumborum* (LL) muscle and decrease in *Semimembranosus* (SM) muscle (in SP2 −41.36% than controls). The α tocopherol and retinol content were enhanced in both muscles of rabbit fed the natural mixture (SP1 and SP2 groups). An improvement of sensory attributes of texture was observed in both muscles from rabbit fed natural mixture. In conclusion, long term supplementation of both lactating does and offspring with the high dosage of brown seaweed and plant polyphenols improves growth performance and enhances meat nutritional and sensory parameters.

## 1. Introduction

The EU is the second highest world producer of meat rabbits. The majority (83%) of production is in Spain (48.5 million rabbits slaughtered), followed by France (29 million rabbits slaughtered) and Italy (24.5 million rabbits slaughtered). Rabbit meat represents a traditional dietary practice in the Mediterranean area and a new source of consumption in China and Mexico [1,2,3].

Even if the global rabbit meat consumption per capita is low if compared with other meat (0.19 kg/per capita/year), in EU countries the consumption is about 0.51 kg/per capita/year (Spain 1.09 kg; Italy 0.91 kg; France 0.75 kg) [3].

In the last decades, the genetic selection has turned towards improvement of the offspring number, growth rate and muscle mass. This causes some problems related to global farm efficiency [4,5]. In rabbit farming several diseases occur; digestive disorders are the main problem, affecting antibiotics consumption [6].

Considering that the EU has been committed for years towards a rational use of antibiotics in livestock, nutrition can be one potential efficient strategy for improving rabbit health and meat quality. Additional research that explores the effects of dietary sustainable additives and alternative feed ingredients in rabbit are required.

Moreover, in recent years, the demand for healthy foods that respect the environment and animal welfare has increased [7]. For products of animal origin, the idea of healthy and safe is linked not only to the product nutritional composition but also to the diet, additives, and antibiotics that the animals have taken. In fact, a strong consumers’ preference for natural food, free from synthetic additives and antibiotics was observed [8].

Several studies reported that dietary integration with plant extracts containing polyphenols in rabbit improves health, increases nutrient digestibility and growth performances and enhances meat quality parameters [9,10]. Plant extracts offer a considerable range of activities such as antioxidant, anti-inflammatory, antiviral and antimicrobial effects, positively influencing feed digestibility and microbial ecology [6].

Other studies showed that dietary brown seaweeds in livestock had a positive effect on health, growth performance, and meat quality due to their nutraceuticals properties and content of sulfated polysaccharides, phlorotannins, diterpenes, omega-3 polyunsaturated fatty acids, minerals and vitamins [11,12]. Moreover, brown seaweeds represent a renewable and sustainable feed ingredient for their high productivity if compared to other conventional ingredients.

Our previous study reported the effects of a mixture of brown seaweeds and plant polyphenols on the performance of lactating does [13]. Moreover, we also investigated the effect of the same natural supplementation in growing rabbits [14]. In literature, no previous study reported the effects of long term supplementation in rabbit with brown seaweed and plant polyphenols from lactating does to rabbit slaughter. Therefore, we wanted to evaluate if dietary treatments during the entire rabbit rearing period may positively affect the growing rabbit’s productive performance and meat quality parameters.

## 2. Materials and Methods

### 2.1. Animals and Dietary Treatments

Rabbits were handled following the guidelines of EU Directive 2010/63/EU and national guidelines for the care and use of animals [15]. All experimental procedures involving animals were approved by the National Agricultural and Food Centre ethical committee (No. NPPC 18-10-2016).

The dietary supplementation began in the lactation phase in which does (n = 60) were divided into three experimental groups, homogeneous for body weight (4.83 ± 0.19 kg) and parity order (second), receiving a control diet (C) or diets supplemented with 0.3% (SP1) and 0.6% (SP2) of feed additive containing prebiotic polysaccharides from brown seaweeds (*Laminaria* spp.) and plant extracts containing phenolic acid, hydroxycinnamic acids, tannins, and flavonoids. In the postweaning period, all rabbits remained in the treatment group defined by their does. The trial lasted 42 days.

The dosages of the supplement were chosen based on an in vitro study on the minimal inhibitory concentration (MIC) against *Clostridium* spp., *Staphylococcus* spp., and *Escherichia coli* spp. [16].

No anticoccidials, antibiotics or other medications were included in the diet. The feed additive was included in the mashed diets, then the diets were pelleted.

At weaning (35 days), 144 New Zealand White rabbits, balanced for body weight and sex were housed at the National Agricultural and Food Centre, Nitra (Slovak Republic).

Rabbits were randomly allotted into three experimental groups (48 rabbits per treatment) and were housed in cages (2 rabbits/cage). The cages were equipped with a hopper for feed and an automatic nipple drinking system. The lighting cycle throughout the trial was 16 h of light and 8 h of dark. The building temperature was maintained within 18 ± 4 °C, with a relative humidity of 70 ± 5%.

The animals were fed ad libitum and had free access to water. Table 1 reports the ingredients and chemical composition of the experimental diets.

The chemical composition was analyzed according to the methods of the Association of Analytical Chemists [17], following the recommendations of the European group on rabbit nutrition [18].

Table 2 reports the chemical composition, phenolic composition and carotenoid content of the feed supplement. The supplement phenolic compounds were determined using HPLC-UV-DAD [19]. The beta-carotene content of the feed supplement was performed according to Rakusa et al. [20].

### 2.2. Sampling

The animals were weighed at 0 and 42 days of the experimental trial and were monitored daily to assess the health conditions. Daily feed intake was recorded (feed offered and refused weekly). The data were used to calculate the average daily gain (ADG), average daily feed intake (ADFI) and feed conversion ratio (FCR).

At the end of the trial the rabbits (77 days old) were weighted and after a fasting period of 6 h 1 male rabbit per cage (12 animals/group) were randomly selected and slaughtered. Rabbits were subjected to electrical stunning (100 V, 50 Hz, 2–3 s) and sacrificed by bleeding according to the guidelines established by the European Community (1099/2009/EC) for the protection of animals during slaughter [21].

Carcasses were chilled for 24 h at +4 °C and then dissected, according to the recommendations of the WRSA [22], by removing the skin, distal part of the limbs, genital organs, bladder and the gastrointestinal tract. Carcasses were then weighted, and the dressing percentages were determined. Samples of whole *Longissimus lumborum* (LL) from the 7th thoracic vertebra to the 7th lumbar vertebra and whole thighs were collected. The physical parameters were evaluated on freshly cut LL muscle and *Semimembranosus* (SM) muscle. The whole muscles were then sampled for chemical and sensory analysis, vacuum packed and stored at −20 °C. The chemical parameters were performed on LL and SM muscles and the sensory evaluation was assessed on the LL muscle and whole thighs.

### 2.3. Physical and Chemical Parameters

All the analyses were performed on LL muscle and *Semimembranosus* (SM) muscle. The pH and color parameters were measured 24 h after slaughter. The pH measurement was performed using a pH meter (HI98191 microcomputer; Hanna Instruments, Vila do Conde, Portugal), calibrated with a standard buffer of pH 4.0 and 7.0.

Meat color parameters were evaluated at 24 h after slaughter on the fresh cut surface of LL and SM muscles, after 20 min of blooming, using a chromameter (Chromameter CR 300 Minolta Ltd., Osaka, Japan) equipped with a D65 light source and a 0° viewing angle geometry according to the reflectance coordinates (L*, a*, b*). The instrument was calibrated using a white calibration plate (Calibration Plate CR-A43; Minolta Camera Co.). The color determination was assessed in triplicate.

The chemical composition of both muscles was determined according to the AOAC methods [17].

The cholesterol content was determined following the method of Du and Ahn [23], using a gas chromatographic method. The cholesterol was identified based on the retention time of the standard (Sigma Aldrich, St. Louis, CA, USA) and quantified with the Chrom Card Data System (version 1.17) software. All samples were analyzed in triplicate.

### 2.4. Vitamin Content

The α-tocopherol and retinol content in LL and SM muscle were determined using the modified procedure of Zaspel and Csallany [24]. The samples were analyzed using an HPLC system (Kontron Instruments, Milan, Italy) with an autosampler (HPLC autosampler 360, Kontron Instruments, Milan, Italy) with a loop of 20 μL, a high-pressure pump and a C18 column 5 μm, 150 × 4.6 mm (Phenomenex, Torrance, CA, USA). The mobile phase was acetonitrile and methanol (75:25 *v*/*v*) and the flow rate was 1 mL per min. The α-tocopherol and retinol were identified using a fluorimeter detector, comparing the retention time with the standards (Sigma Aldrich, St. Louis, CA, USA). The quantification was performed using the Geminyx system (version 1.91).

### 2.5. Sensory Analysis

The sample preparation was performed after thawing thighs and LL muscle for 24 h at 4 °C. The whole samples of LL and thighs were prepared in an uncovered stainless-steel dish in a conventional oven (REX, Milano, Italy) at 180 °C. A thermocouple (Pentronic AB, Gunnebobruk, Sweden) was used to register the sample core temperature and samples were removed at 79–80 °C. The roasted samples were then cut into 1.5 cm thick slices (Electrolux 50, 220–24, kW0.2) and the slices were warmed to 60 °C before the evaluation. A trained sensory panel, involving eight members familiar with descriptive analysis procedures [25], was employed. All evaluations were performed in a sensory laboratory equipped according to EN ISO 8598 recommendations [26]. The descriptors, definitions, and standards are reported in Palazzo et al. [27].

The sensory profile was assessed according to EN ISO 13,299 [25] and the panel evaluated the two samples (thigh and LL) on different days in triplicate. Within each session the design was balanced for order and carry-over effects [28]. During training and sampling, the panelists had access to unlimited water and unsalted crackers. They were requested to evaluate the intensity of each attribute by assigning a score between 1 (absence of the sensation) and 9 (extremely intense).

### 2.6. Statistical Analysis

Data were analyzed using SPSS software (SPSS, PC Statistics 25.0 SPSS Inc., IBM, Segrate, MI, Italy). Productive performance and slaughter and meat quality parameters were analyzed using one-way analysis of variance (ANOVA), with diet as the fixed effect. The sensory data were submitted to ANOVA with samples, judges, replicates, and their interactions as effects (EN ISO 13299, 2010). The significance of these effects was tested with F tests. Post-hoc pairwise contrasts were evaluated by Duncan’s test. The cage was considered as the experimental unit for growth performance and the rabbit for the meat quality parameters. Data are reported as mean ± SEM. Differences among treatments were considered significant at *p* < 0.05.

## 3. Results

### 3.1. Productive Parameters and Carcass Characteristics

The growth performances and carcass parameters of rabbit fed experimental diets are reported in Table 3. The final weight of the rabbits did not differ among experimental groups (*p* > 0.05), even if the ADG tended to be higher (*p* = 0.09) in both the SP1 and SP2 groups. The ADFI result was lower (*p* < 0.05) in the SP1 group than the results in the other groups. The feed conversion ratio was positively affected (*p* < 0.001) by both the dosage of brown seaweed and plant polyphenols (SP1 and SP2).

The slaughter weight (2791.3 ± 69.2 g C vs. 3001.2 ± 59.4 g SP1 vs. 2986.1 ± 42.4 g SP2; *p* = 0.021) and the carcass weight (1473.4 ± 38.6 g C vs. 1585.2 ± 33.4 g SP1 vs. 1610.4 ± 19.7 g SP2; *p* = 0.010) of the sampled rabbit (n = 12) resulted higher in groups fed brown seaweed and polyphenols (SP1 and SP2) than in the control. The dressing percentage resulted higher in SP2 group than the others (52.8 ± 0.37% C vs. 52.4 ± 0.32% SP1 vs. 53.9 ± 0.41% SP2; *p* = 0.025).

### 3.2. Meat Physical and Chemical Parameters

The chemical composition of LL and SM muscle are reported in Table 4 and Table 5, respectively. The physical parameters in both muscles were not affected (*p* > 0.05) by long term supplementation of both lactating does and the offspring with low and high dosage of seaweed and polyphenols. The nutritional parameters moisture, crude protein, ether extract and ash were also unaffected by dietary treatments (*p* > 0.05). The cholesterol content in LL muscle tended to be lower (*p* = 0.059) in SP2 than SP1 and the control groups. The cholesterol content of the SM muscle resulted lower (*p* < 0.01) in the group that received the high dosage of seaweed and polyphenols (SP2) than SP1 and the control groups. The cholesterol content reduction was about −40.3% than control and −36.4% than SP1 groups.

### 3.3. Muscle Vitamin Content

**Figure 1:**
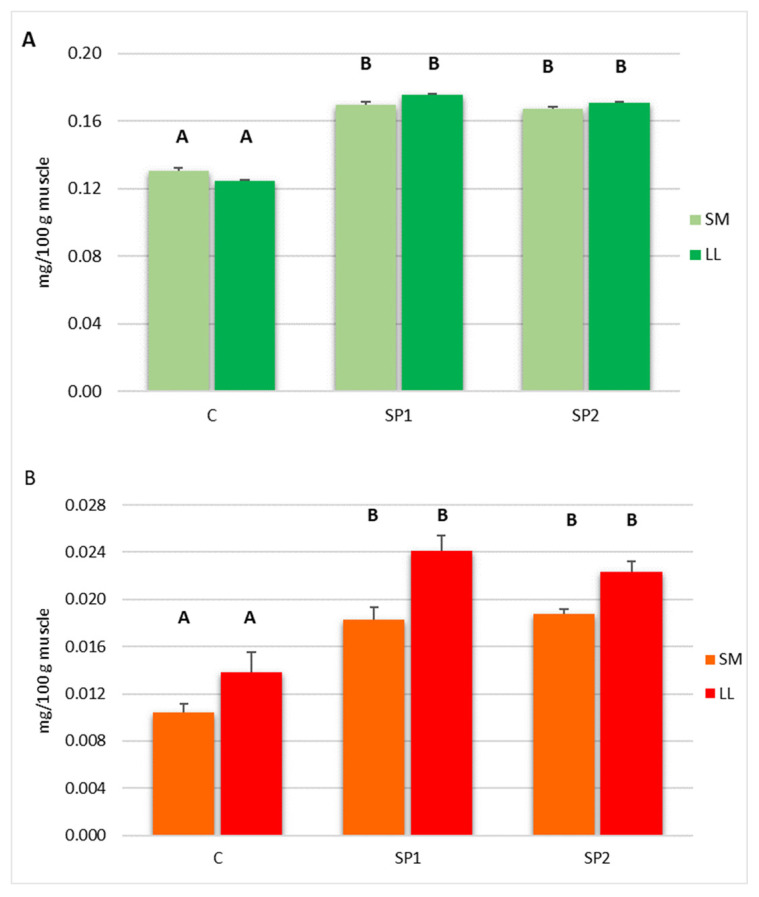
α-tocopherol (**A**) and retinol (**B**) content of *Longissimus lumborum* (LL) and *Semimembranosus* muscle (SM) of rabbits fed control diet (C) and diet supplemented with 3 g/kg (SP1) and 6 g/kg (SP2) of brown seaweed and plant polyphenols. Data are reported as means ± standard error of the means (SEM); n = 12. ^A,B^ values with different superscript letters are different at *p* < 0.001.

Figure 1 shows the α-tocopherol (A) and retinol (B) content of both muscles in relation to dietary treatments. The α-tocopherol content resulted higher (*p* < 0.001) in the LL and SM muscles of rabbit fed both dosages of brown seaweed and polyphenols (SP1 and SP2) than in the control. The retinol content was higher (*p* < 0.001) in both muscles of rabbit fed brown seaweed and plant polyphenols (SP1 and SP2) than in the control group.

### 3.4. Sensory Profile

The F values for the measured parameters of the LL muscle and thigh sensory profile are reported in Table 6 and Table 7, respectively. The data show that dietary supplementation with brown seaweed and plant polyphenols mixture affected (*p* < 0.05) the flavor and texture of the LL muscle. The F values for replicates and interactions were not affected (*p* > 0.05) for all the descriptors. In thigh, taste, flavor and texture were affected (*p* < 0.05) by dietary treatments. Judges presented differences (*p* < 0.05) for aroma, taste, flavor and texture as observed for LL muscle. The judges and the interaction of the treatments per judge revealed differences (*p* < 0.05) for aroma, taste, flavor and texture.

**Figure 2:**
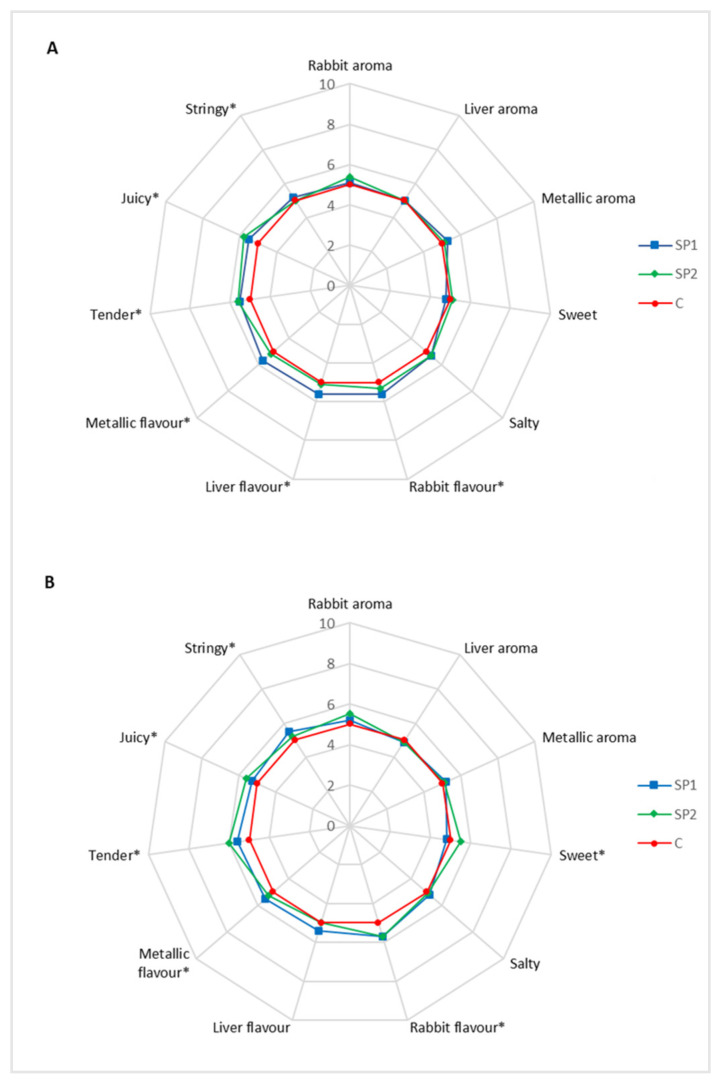
Spider plot of the sensory profile of *Longissimus lumborum* muscle (**A**) and thigh (**B**) of rabbit fed the control diet (C) and the diet supplemented with 3 g/kg (SP1) and 6 g/kg (SP2) of brown seaweed and plant polyphenols. * Significant at *p* < 0.05.

Figure 2 reports the least squares mean of the different attributes for the LL muscle (A) and thighs (B). In LL muscle the rabbit, liver and metallic flavors were affected by the high dosage of brown seaweed and plant polyphenols, resulting higher (*p* < 0.05) in the SP2 group than the SP1 group and the control groups. The texture parameters were also enhanced in muscle from rabbit fed the natural mixture. In particular, tenderness and juiciness resulted higher (*p* < 0.05) in both groups receiving brown seaweed and plant polyphenols (SP1 and SP2) than the controls. The stringiness resulted lower (*p* < 0.05) in the control and SP2 groups than results in the SP1 group. The attributes related to aroma and taste were unaffected (*p* > 0.05) by dietary treatments. In tights the rabbit and metallic flavors were affected by brown seaweed and plant polyphenols, resulting higher (*p* < 0.05) in both groups receiving the supplement (SP1 and SP2) than in the controls. The sweety taste was positively affected (*p* < 0.05) by the high dosage of natural supplement, in fact, thigh from SP2 group resulted sweetness higher than controls. Tenderness and juiciness resulted higher (*p* < 0.05) in groups receiving the high dosage of brown seaweed and plant polyphenols (SP2) than the SP1 group and the controls. The stringiness resulted lower (*p* < 0.05) in the control group than the SP1 group. The attributes related to aroma were unaffected (*p* > 0.05) by dietary treatments.

## 4. Discussion

In the recent years, several studies reported the effect of dietary supplementation with plant polyphenols in rabbit to improve growth performances and meat quality parameters [9,29]. However, there are only few studies on dietary supplementation with seaweed in rabbit. Previous study reported that dietary green seaweed *Ulva Lactuca* in does and male rabbit improve reproductive parameters [30,31]. Moreover, Dalle Zotte et al. [32] reported that a mixture containing dietary microalga *Spirulina* and *Thymus vulgaris*, did not affect growth performance and energy or nutrients digestibility.

To the best of our knowledge, the first study describing the effect of dietary brown seaweed and plant polyphenols in rabbit is our recent study limited to the growing phase [14], but the effects of long term supplementation of this mixture from lactating does to rabbit slaughter has been not yet studied. The present data showed that ADG tended to be higher in rabbit fed the natural mixture and the feed conversion ratio was enhanced in rabbit fed the dietary supplement, suggesting a better feed conversion due to the probiotic, antibacterial and antioxidant effects of the natural mixture used [11]. An enhancement of nutrient digestibility was also observed in several animal species fed seaweed [12]. The present data are in line with studies in rabbit showing that natural extract dietary supplementation positively affected rabbit’s growth performances [6,10].

The data on carcass characteristics showed an enhancement in rabbit fed the natural dietary supplement. Previous studies reported no effects of dietary supplementation with polyphenols on carcass characteristics in pigs and rabbit [9,33]. Other studies in pigs reported an enhancement of carcass characteristics due to polyphenols supplementation [34,35]. These different results are probably due to the length and dosage of dietary supplementation and the active principles content of the plant extract used.

The present data show that dietary supplementation with polyphenols and brown seaweed did not affect pH and color parameters in both muscles, in agreement with our previous study in rabbit fed the same mixture [14]. The data fall in the means of the values reported in other studies for LL and SM muscles [36,37].

Likewise, the chemical composition of the LL and SM muscles was not affected by the dietary treatments, except for the cholesterol content. Comparing with our study in rabbit fed the same mixture [14], the dietary supplementation of both the does and the offspring reduces the cholesterol content in both muscles, and this reduction compared to controls was relevant in the SP2 groups (−24.5% and −40.3% in LD and SM muscle, respectively).

Previous studies reported a lowering cholesterol effect of seaweed in livestock and humans. Al-Harthi and El-Deek [38] reported that dietary supplementation with 3–6% of brown seaweed in laying hens improved egg quality, and decreased yolk cholesterol content. In humans, seaweed intake was positively linked to a modulation of blood glucose and cholesterol [39]. This mechanism is probably linked to the activity of the seaweed polysaccharide fucoidan that decreases cholesterol absorption and increases its excretion, modulating reverse cholesterol transport-related protein expression [40]. A cholesterol lowering effect was also observed in rabbit and hares fed plant extract containing polyphenols [41,42].

Our results show that the dietary mixture of brown seaweed and plant polyphenols enhances the content of α tocopherol and retinol in both LL and SM muscles. Seaweeds show antioxidant properties due to the phenols, carotenoid fucoxanthin, tannins, phlorotannins and polysaccharides content. Furthermore, seaweeds are an excellent source of vitamins and, above all, brown seaweeds contain a large amount of α tocopherol and carotenoid [11]. In fact, previous studies in pork reported that seaweed dietary supplementation enhances oxidative stability of LD muscle due to higher muscle α tocopherol content [43,44].

Moreover, plant extract containing polyphenols increased α tocopherol and retinol levels in several tissues, reducing the oxidative markers [45]. In fact, an earlier study in pork, rabbit and hares reported that natural extract containing polyphenols enhanced muscle α tocopherol and retinol [27,46,47].

The data on sensory profile revealed that the dietary mixture of brown seaweed and plant polyphenols affected the flavor in both LL muscle and thigh, even if the differences are hardly perceived. The texture attributes related to tenderness, juiciness and stringiness was improved in in both LL muscle and thigh from rabbit fed the natural mixture. The judges and the interaction of the treatments per judges revealed differences for aroma, taste, flavor and texture, but this is common in sensory evaluations, due to the different use of the scale [48]. Even if there is selection and training of judges, some variability always remains, but the interaction treatments per judges was not significant for most descriptors, indicating low variability among the panel members.

In literature no previous study reported the effects of dietary brown seaweed supplementation on meat sensory quality parameters while several studies reported the effects of dietary plant polyphenols on this parameter. Dietary plant polyphenols from *Lippia* spp. improve the texture parameters related to tenderness and juiciness in donkey and horse *Longissimus dorsi* muscle and it is probably related to the protection against the oxidation process [27]. Moreover, Zhao et al. [49] also reported an improvement of tenderness of lamb meat due to dietary supplementation with wine grape pomace, a rich source of polyphenols. Probably, the protection of the proteolytic enzymes μ-calpain and m-calpain from the oxidative process, increases their functionality, enhancing meat tenderness [50,51].

## 5. Conclusions

Our results show that dietary supplementation with a high dosage of brown seaweeds and plant polyphenols of both lactating does and the offspring have a positive effect on the average daily feed intake and feed conversion ratio, without affecting other meat physical parameters.

Nutritional characteristics of both LL and SM muscle were positively affected by the natural extract, with a reduction of the cholesterol content and an increase of α tocopherol and retinol content. Moreover, an improvement of the sensory quality perceived by consumers in terms of texture was observed in both LL muscle and thigh from rabbit fed the high dosage of the natural mixture.

From these data, we can conclude that the long term supplementation of both lactating does and the offspring with a high dosage of brown seaweeds and plant polyphenols is a valid approach to boost rabbits’ performance and enhance meat nutritional and sensory parameters.

## Figures and Tables

**Table 1:** Ingredients (g/kg) and chemical composition of experimental diets.

Ingredients	C	Diet ^a^ SP1	SP2
Maize	282	279	276
Alfalfa hay	305	305	305
Sunflower meal	135	135	135
Carob bean meal	90	90	90
Wheat	80	80	80
Oat	53	53	53
Cane molasses	20	20	20
Palm seed oil	8	8	8
Soybean oil	7	7	7
Calcium carbonate	7	7	7
Sodium Chloride	3	3	3
DL-Methionine (99%)	2.5	2.5	2.5
Vitamin and mineral premix ^b^	2.5	2.5	2.5
Dicalcium phosphate	2	2	2
L-Lysine HCl (78.5%)	1.6	1.6	1.6
Choline (75%)	1.4	1.4	1.4
Dietary supplement	0	3	6
Chemical composition ^c^			
Crude protein	184	183.6	183.5
Ether extract	37.5	35.5	35.5
Crude fiber	187	186.8	187
Ash	86	85.7	85.8
Nitrogen free extract	507	507.1	506.9
NDF	302.1	301.5	301.7
ADF	195.8	195.4	195.3
ADL	39.9	39.5	39.5

^a^ C = control diet; SP1 = diet supplemented with 0.3% of brown seaweed and plant polyphenols; SP2 = diet supplemented with 0.6% of brown seaweed and plant polyphenols. ^b^ Supplied per kg diet: 13,500 I.U. vitamin A (trans-retinyl acetate); 800 I.U. vitamin D3 (cholecalciferol); 35 mg vitamin E (α-tocopherol min 91%), 35 mg copper (cupric sulphate pentahydrate). ^c^ Determined in triplicate.

**Table 2:** Chemical composition and polyphenols content of the dietary supplement.

Item	
	% DM
Dry matter	93.6 ± 5.05
Crude protein	7.2 ± 0.99
Ether extract	0.32 ± 0.01
Crude fiber	11.2 ± 1.02
Carbohydrates	49.6 ± 3.18
Ash	32.7 ± 1.38
Chemical compounds: ^a^	mg/kg DM
β-Carotene	402 ± 30.89
Phenolic acid:	
Syringic acid	1059.8 ± 62.82
Hydroxycinnamic acids:	
Neochlorogenic acid	7979.2 ± 468.11
Rosmarinic acid	126.5 ± 8.67
Trans sinapic acid	105.5 ± 8.09
Chlorogenic acid	21.4 ± 3.65
Tannins:	
Ellagic acid	2440.9 ± 148.29
Rutin	272.4 ± 20.82
Flavonoids:	
Myricetin	53.9 ± 5.68

^a^ Values are expressed as means (n = 4) ± standard deviation.

**Table 3:** Growth performances of rabbits fed the control diet (C) and diet supplemented with 3 g/kg (SP1) and 6 g/kg (SP2) of brown seaweed and plant polyphenols.

Item	C	Diet SP1	SP2	*p*-Value
Initial weight, g	829.1 ± 0.21	838.7 ± 0.22	818.8 ± 0.17	0.686
Final weight, g	2657.2 ± 58.62	2757.9 ± 41.54	2864.1 ± 35.51	0.220
ADG, g/d	42.5 ± 1.16	45.7 ± 1.04	46.3 ± 0.73	0.090
ADFI, g/d	172.1 ± 2.45 ^a^	148.8 ± 3.17 ^b^	161.9 ± 1.66 ^a^	0.014
FC	3.95 ± 0.04 ^A^	3.25 ± 0.06 ^B^	3.49± 0.03 ^B^	0.001

ADG, average daily gain; ADFI, average daily feed intake; FC, feed conversion ratio. Data are reported as means ± standard error of the means (SEM); n = 48 ^a,b^ values in the same row are different at *p* < 0.05. ^A,B^ values in the same row are different at *p* < 0.01.

**Table 4:** Physical and chemical parameters of *Longissimus lumborum* muscle of rabbits fed the control diet (C) and diet supplemented with 3 g/kg (SP1) and 6 g/kg (SP2) of brown seaweed and plant polyphenols.

Item	C	Diet SP1	SP2	*p*-Value
pH 24 h	5.85 ± 0.25	5.91± 0.21	5.88 ± 0.16	0.222
Color parameters				
L*	55.85 ± 0.39	56.30 ± 1.01	56.44 ± 0.81	0.873
a*	3.89 ± 0.71	3.99 ± 0.32	4.28 ± 0.42	0.911
b*	11.92 ± 0.67	11.00 ± 0.52	11.28 ± 0.26	0.484
Chemical composition:				
Moisture, %	73.23 ± 0.18	73.02 ± 0.21	72.84 ± 0.18	0.383
Crude Protein, %	24.33 ± 0.14	24.52 ± 0.17	24.65 ± 0.21	0.404
Ether extract, %	1.17 ± 0.06	1.05 ± 0.06	1.19 ± 0.10	0.547
Ash, %	1.22 ± 0.01	1.36 ± 0.08	1.39 ± 0.06	0.132
Cholesterol, mg/100 g	33.23 ± 2.98	28.18 ± 1.24	25.07 ± 1.18	0.059

Data are reported as means ± standard error of the means (SEM); n = 12.

**Table 5:** Physical and chemical parameters of *Semimembranosus* muscle of rabbits fed the control diet (C) and diet supplemented with 3 g/kg (SP1) and 6 g/kg (SP2) of brown seaweed and plant polyphenols.

Item	C	Diet SP1	SP2	*p*-Value
pH 24 h	5.77 ± 0.20	5.79± 0.18	5.82± 0.21	0.853
Color parameters				
L*	63.92 ± 1.59	63.84 ± 2.01	64.41 ± 1.94	0.951
a*	6.01 ± 0.64	5.94 ± 0.56	6.11 ± 0.74	0.894
b*	3.92 ± 0.32	4.02 ± 0.49	3.78 ± 0.56	0.831
Chemical composition:				
Moisture, %	74.29 ± 0.86	74.22 ± 0.78	74.17 ± 0.72	0.594
Crude Protein, %	23.12 ± 0.53	23.37 ± 0.73	23.61 ± 0.62	0.338
Ether extract, %	1.66 ± 0.46	1.92 ± 0.38	1.51 ± 0.43	0.958
Ash, %	1.21 ± 0.06	1.19 ± 0.09	1.15 ± 0.07	0.329
Cholesterol, mg/100 g	39.85 ± 4.48 ^A^	37.39 ± 2.07 ^A^	23.78 ± 1.17 ^B^	0.002

Data are reported as means ± standard error of the means (SEM); n = 12. ^A,B^ values in the same row are different at *p* < 0.01.

**Table 6:** The F value and statistical significance of treatments (C, SP1, SP2) and their interaction for sensory descriptors of *Longissumus lumborum* muscle, where the number of judges, n = 8, and the number of replicates, n = 3.

Descriptors	Treatments	Judges	F Value Replicates	T*J	T*R	J*R
Aroma						
Rabbit	2.27	4.52	0.26	1.68	1.55	0.85
Liver	0.05	1.73	1.37	0.70	1.75	1.03
Metallic	2.27	4.83 **	0.08	1.63	1.41	0.83
Taste						
Sweet	0.71	2.67 *	1.33	1.64	0.46	1.23
Salty	2.12	2.83 *	0.63	1.10	1.16	1.22
Flavor						
Rabbit	4.61 *	1.37	0.73	0.95	2.25	1.45
Liver	12.88 ***	5.56 ***	0.30	3.41 **	0.77	1.47
Metallic	5.18 *	5.83 ***	0.45	2.19 *	0.30	1.00
Texture						
Tender	8.53 **	7.80 ***	0.81	2.98 **	0.48	1.12
Juicy	20.31 ***	2.69 *	2.04	3.84 **	1.00	1.12
Stringy	3.56 *	3.26 *	1.04	4.83 ***	1.48	0.77

*** *p* < 0.0001; ** *p* < 0.001; * *p* < 0.05.

**Table 7:** The F value and statistical significance of treatments (C, SP1, SP2) and their interaction for sensory descriptors of thigh, where the number of judges, n = 8, and the number of replicates, n = 3.

Descriptors	Treatments	Judges	F Value Replicates	T*J	T*R	J*R
Aroma						
Rabbit	1.91	2.58 *	0.09	1.02	1.28	0.80
Liver	0.11	1.35	1.15	0.65	1.07	1.09
Metallic	035	5.68 **	0.89	2.07 *	0.56	1.10
Taste						
Sweet	3.97 *	0.70	2.90	1.29	2.42	0.81
Salty	0.36	4.06 **	1.02	2.18 *	0.37	0.71
Flavor						
Rabbit	6.51 **	3.26 *	1.22	1.26	0.58	2.42
Liver	2.66	2.46 *	1.66	1.47	1.42	1.43
Metallic	2.97 *	5.09 ***	0.95	1.84	1.13	1.17
Texture						
Tender	16.95 ***	4.44 **	0.93	5.23 ***	0.97	0.46
Juicy	12.92 ***	1.47	0.57	4.92 ***	0.86	0.71
Stringy	7.45 **	14.44 ***	0.39	9.57 ***	0.39	0.84

*** *p* < 0.0001; ** *p* < 0.001; * *p* < 0.05.
